# Relational vulnerability in motherhood—an existential perspective on pain and exhaustion among women

**DOI:** 10.3389/fpsyg.2024.1412385

**Published:** 2024-07-12

**Authors:** Anja J. Gebhardt, Susanne Andermo, Maria Arman

**Affiliations:** ^1^Karolinska Institutet, Department of Neurobiology, Care Sciences and Society, Huddinge, Sweden; ^2^The Swedish School of Sport and Health Sciences, Department of Physical Activity and Health, Stockholm, Sweden

**Keywords:** suffering, chronic pain, burnout, rehabilitation, caring science, existential health, parenting

## Abstract

**Introduction:**

Stress-related ill-health like pain and exhaustion are demanding public health problems in Europe. In Sweden, women are particularly at risk to develop stress-related ill-health during a period in life that coincides with child-rearing years. When entering motherhood, Swedish women’s sick leave substantially increases. Yet, motherhood is rarely acknowledged in clinical encounters concerning pain and exhaustion although women suffer from these ailments more often than men. To incorporate motherhood as an existential dimension of health in the care of women living with pain and exhaustion might alleviate women’s suffering. But knowledge on women’s experiences of motherhood and health is scarce. Therefore, the aim of the study is to reach a deeper understanding of how women suffering from long-lasting pain and exhaustion experience their health in relation to motherhood.

**Methods:**

Ricoeur’s interpretation theory has been applied to analyze 27 phenomenological interviews with 14 mothers suffering from long-lasting pain and exhaustion.

**Results:**

These women’s experiences shed light on how closely motherhood is interwoven with the experience of their health and suffering: The women’s suffering seems to be rooted in a *relational vulnerability* that has been uncovered during motherhood. Further, the women suffer from a burden of *difficult life experiences* and *inner conflicts*. *Reconciliation with life* is possible when women find an *existential shelter*, which offers ways to relate to their suffering making the own suffering more bearable.

## 1 Introduction

Pain related to musculoskeletal disorders and stress-related ill-health such as exhaustion are demanding public health problems in Sweden (Swedish Insurance Agency, 2020; Statistics Sweden, 2023) and across other European countries (Aumayr-Pintar et al., 2018; Todd et al., 2019; Zimmer et al., 2022). Long-lasting pain is noticeably more common among women than men, and women tend to be more exhausted than men (Marchand et al., 2018; Casale et al., 2021; LoMartire et al., 2021). Stress has been suggested as having both a protective and damaging effect on long-lasting pain, but the stress level for which this sweet spot of protection occurs is yet unknown (Lunde and Sieberg, 2020). In Sweden, women are particularly at risk of developing stress-related ill-health such as exhaustion during a period in life that coincides with child-rearing years (Swedish Insurance Agency, 2020). When entering motherhood, Swedish women’s sick leave substantially increases, and this effect persists for almost two decades throughout a woman’s life (Angelov et al., 2020). Usually, affected women seek care for many years without being properly helped; further, they often feel their suffering is not taken seriously, which leads to further suffering (Samulowitz et al., 2018; Arman et al., 2020). Available treatments for pain and exhaustion, such as multimodal rehabilitation, have been found to provide only modest effects or have been considered insufficient, though they have been found to be an improvement over routine care (Kamper et al., 2015; Wallensten et al., 2019; Pietilä-Holmner et al., 2020). Further, the prevailing biomedical and biopsychosocial models have been criticized as fragmented and overly narrow in the encounter with individuals suffering from long-lasting pain (Toye et al., 2013; Stilwell and Harman, 2019). Both Stilwell and Harman (2019) and Toye et al. (2013) argue for an understanding of pain that acknowledges pain as a first-person experience that is inseparable from a person’s unique relations with the world and the meaning connected to these relations. There is a vast body of knowledge on how women’s pain impacts their children and family functioning (Higgins et al., 2015), but exploration of women’s subjective experience of their health in relation to motherhood has been scarce, although there is a growing interest for exhaustion related to parenthood (Roskam et al., 2021).

Although there is evidence that considering the gender context helps to explain variations in pain between the sexes, empirical gaps have been identified by Keogh (2021). Specifically, the relation between gender identity and the development of pain across the life course has been proposed to be one important aspect to be explored (Keogh, 2021). In the current study, motherhood is regarded to be one important—although not defining—aspect of gender identity that comes with substantial changes and challenges in the life course of a feminine identity. Becoming and being a mother is something most women will experience and involves a life-long commitment that impacts women’s health in various ways (Graham, 2015; Duarte-Guterman et al., 2019). Intense periods of childcare as represented by a cohort of twin mothers were shown to be correlated with stress-related mortality in old age (Bucher-Koenen et al., 2020). This suggests that women might be strained for a long time when combining motherhood and working life (Angelov et al., 2020; Bucher-Koenen et al., 2020; Swedish Insurance Agency, 2020). Nevertheless, motherhood is rarely acknowledged as a relevant aspect in clinical healthcare encounters when women seek care for reasons other than reproductive health. Thus, in the care of women suffering from these conditions, a broader perspective on health that takes women’s specific life circumstances into account is needed. Hence, the aim of the study was to reach a deeper understanding of how women suffering from long-lasting pain and exhaustion experience their health in relation to motherhood.

## 2 Materials and methods

### 2.1 Study design

The study’s methodological approach was based on Paul Ricoeur’s interpretation theory (Ricoeur, 1976, 1981). Ricoeur elaborated on Gadamer’s phenomenology of understanding (Gadamer, 2004) by introducing *explanation* as a counterpart to *understanding* (Ricoeur, 1976, 1981). According to Ricoeur, explanation and understanding are both contradictory and mutually dependent in the process of interpretation. Ricoeur emphasizes the importance of method or structural analysis, which he regards as intertwined with the epistemology of interpretation. Ricoeur considered “structural analysis as a stage – and a necessary one – between a naïve interpretation and a critical interpretation” (Ricoeur, 1981, p. 218) and located “explanation and understanding at two different stages of a unique *hermeneutical arc*” (Ricoeur, 1981, p. 218). To construe the meaning of a text implies looking at both the parts and the whole and their relationship, which is a process that is circular in nature. This process refers to the hermeneutical circle as a concept of validation in which the most probable interpretation is screened. In short, explanation is equivalent to the concept of validation, whereas understanding is equivalent to construing the meaning of a text. Thus, in hermeneutics, explanation and understanding are not mutually exclusive, but interdependent (Ricoeur, 1981). Ricoeur’s philosophy of text and interpretation theory has allowed us to weave the interpretations from single conversations into one common interpretation uniting the various experiences of all participating women. We have been able to work out invariable and meaningful structures that describe how women’s experiences over a lifetime are related to motherhood, health, and experiences of suffering.

The study’s reporting follows the consolidated criteria for qualitative research (COREQ) developed by Tong et al. (2007).

### 2.2 Recruitment and interviewing

Women were recruited at one of Sweden’s largest rehabilitation clinics specialized on exhaustion and long-lasting pain. During the second half of a 10-week rehabilitation program, the women were consecutively asked in person (by AG) to voluntarily participate in two interviews. The aim of the interviews was presented as an exploration of the health and suffering of women with a particular focus on motherhood. The researcher (AG) presented herself as midwife and doctoral student with a particular interest for women’s health, no personal goals with the research were revealed. To be enrolled in the study, women had to have at least one child of any age (adult children included). Thirty-two women expressed their interest in participation and received written information by email, including proposed times for the first interview at the clinic. Out of these, 20 women initially wanted to schedule an interview, but six canceled due to the COVID-19 pandemic.

Fourteen mothers undergoing rehabilitation for exhaustion and/or long-lasting pain were included in the study (for detailed participant characteristics, see Table 1). At the time of the first interview, most participating women had been on sick leave for less than 1 year, and three women had been on sick leave for more than 3 years. Nine women had a partner they either were cohabiting with or married to. Almost half of the women had caring responsibility for a relative (a parent or a sick sibling). The children varied in age from toddlers to adult children. Most of the women became first-time mothers in their twenties, one became a mother late in life and another became a mother as a teenager. The women primarily worked in human service professions (including childcare) or administration. All women had been working fulltime prior to their sick leave. Most lived in the suburbs of Stockholm (both well-situated and less well-situated); only a few women lived directly in Stockholm or just outside Stockholm. All women were born in Sweden.

**Table 1:** Participants’ characteristics (*N* = 14).

Characteristic	Value
Age [years; median (min-max)]	50 (34–63)
Cohabiting partner or married [*n* (%)]	9 (64.3)
Number of children [mode (min-max)]	2 (1–6)
**Mothers with**	
Small children (< 6 years) [*n* (%)]	4 (28.6)
School-age (6–12 years) [*n* (%)]	4 (28.6)
Adolescents (13–17 years) [*n* (%)]	2 (14.3)
Adult children (> 18 years) [*n* (%)]	8 (57.1)
Caring responsibility for other relatives [*n* (%)]	6 (42.9)
**Education**	
Comprehensive school [*n* (%)]	2 (14.3)
High school [*n* (%)]	4 (28.6)
University [*n* (%)]	8 (57.1)
**Total household income (Swedish krona/month)**	
Under 30,000 [*n* (%)]	4 (28.6)
Under 45,000 [*n* (%)]	1 (7.1)
Under 60,000 [*n* (%)]	2 (14.3)
Over 60,000 [*n* (%)]	7 (50.0)
**Sick leave** [*n* (%)]	14 (100)
**Rehabilitation for**	
Exhaustion [*n* (%)]	5 (35.7)
Pain [*n* (%)]	4 (28.6)
Exhaustion and pain [*n* (%)]	5 (35.7)

The women were invited to participate in two interviews: the first interview explored the meaning of motherhood, and the second interview followed up on the first one and explored the experience of health. The interviews were conducted during March and December of 2020. The first interview took place when the women were still in rehabilitation, whereas the second interview was conducted after the rehabilitation period. Half of the interviews were done at the clinic and the other half via video call, depending on what the women preferred. Video calls were offered due to circumstances created by the COVID-19 pandemic. All women but one participated in both interview occasions, yielding 27 recorded interviews ranging from 50 min to 90 min in length. The time between the first and the second interview ranged from 3 to 23 weeks (median 7 weeks). The variations in place and timespan between the interviews were mainly caused by the pandemic; differences in the course and content of the interviews were not observed. No field notes were taken.

Phenomenological interviewing (Hoffding and Martiny, 2016) was chosen to collect the data because it offered an opportunity to work with the women to co-generate an understanding of health and suffering in motherhood. Phenomenological interviews are reciprocal and co-creational in nature; hence, this method heavily relies on an interviewer’s concentration, empathy, and training (Hoffding and Martiny, 2016). In this study, the interviewer (AG) had been trained in interviewing and empathetic conversation. Both interviews were opened with a single question, which was then followed up by previously unseen questions and reflections on experiences shared by the women. The first interview was opened with the question, *“What did, and still does, it mean to you to be a mother?,”* and the second interview with the question, *“How do you experience your health?”* The researcher was careful to formulate reflections exclusively using themes or words that the woman herself had brought up earlier in the interview. This interactive process was particularly suitable because the second interview offered occasions to spontaneously reflect on observed structures in the reported experiences, which often stimulated the women’s own reflections. All interviews were performed, and audio recorded by a single researcher (AG) with no other person present besides the participant and were transcribed by a transcription service. Transcripts were not returned to participants for comments.

### 2.3 Interpretation of data

**Figure 1:**
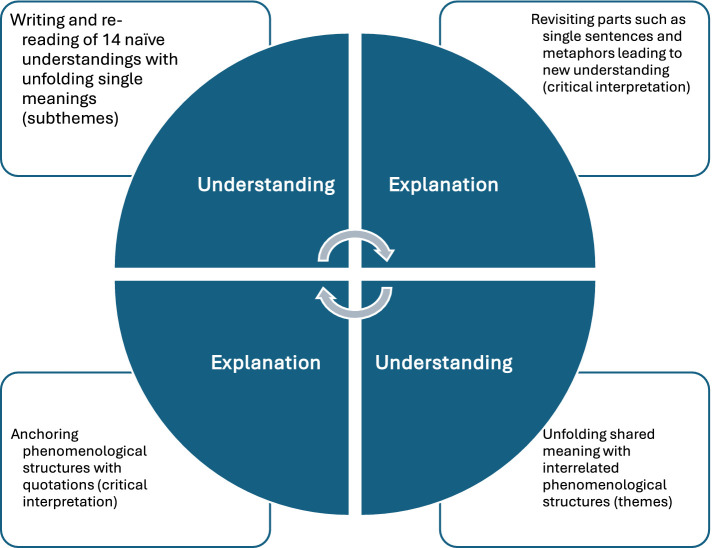
Illustration of the analytical process of constantly moving between understanding and explanation and between naïve and critical interpretation.

We entered a discourse on two levels: in direct conversation with the women, but also when reading the transcribed interviews as texts, independent of the contexts of space and time. As two interviews were planned with each woman, thoughts relating to interpretation that spontaneously arose could be further explored with the women during the second conversation. Ricoeur’s approach to understanding a text’s meaning implies considering the meaning of the words’ relationship with each other, sentences, metaphors, and to see the world that unfolds in front of the text. The unfolding meaning of the two transcribed conversations with each woman was condensed into a single naïve understanding by AG, resulting in one individual interpretation of each woman’s experience. The process of reading, writing, and interpreting each woman’s unique experience revealed a common world of meaning shared by all women. The interrelated structures of meaning that appeared were formulated and re-formulated in several themes as presented in the findings. The research team has reached a consensus on the final structure of the themes. According to Ricoeur (1976), the world of meaning that unfolds in front of a text is inevitably intertwined with all words, sentences, and metaphors within the text. For this reason, we consciously strived to understand the unfolding meaning when reading the text as a whole, but also revisited the parts (single sentences and metaphors) to anchor the most probable interpretation of the text. During this part of the interpretation process, we entered what Ricoeur (1981) described as the *hermeneutical circle*, constantly moving between naïve and critical interpretation (Figure 1). We provide an insight into this process when presenting our *interpretation* of the women’s experiences in the result section by anchoring it in *explanations*, as represented by discrete, embedded, or longer quotations (Eldh et al., 2020). Participants were not asked to provide feedback on the interpretation.

Involved researchers (AG, SA, MA) had different educational backgrounds (ranging from anthropology, public health, and midwifery), and all were women with their own personal experiences of being a mother; internal discussions within the team helped researchers distance themselves from their personal experiences. Qualitative analysis software was not applied to carry out the analysis.

### 2.4 Research ethics

The study was approved by the Swedish Ethical Review Authority (no. 2019–02584). Informants gave their informed and written consent for study participation. The interviews were conducted with a respectful and sensitive attitude. The women were informed that their participation was voluntary and that they could end the interview at any time. No relationship with the participants was established prior to study commencement.

## 3 Results

### 3.1 Summary of the interlaced phenomenological structures

The women suffered from a variety of bodily expressions and *loss of meaning in life*. The women’s suffering seemed to be rooted in a *relational vulnerability*: Being insufficiently embedded in warm and beneficent relational ties risked becoming harmful to the women’s health. In motherhood, it was likely that a relational vulnerability was revealed because being a mother meant being particularly dependent on the support and care of others. Further, interacting with one’s own children could be challenging and often involved confronting memories from difficult life experiences, which in turn could give rise to *strong inner ideals of motherhood* and *inner conflicts*. Facing the demands of being a mother, inner conflicts, and memories of difficult life experiences, all without reliable support and care from others, could be overwhelming and constituted an *unbearable burden*. This burden carried alone gave rise to suffering. Being part of an *existential shelter,* where a woman could be herself and express herself, could relief the unbearable burden and opened the door to *reconciliation with life*. Reconciliation meant letting go of previous experiences, difficult relationships, ideals, and responsibilities, and involved an altered relationship with oneself and one’s life. An evolving awareness of how significant an existential shelter was for one’s own health and suffering, and then nurturing this shelter, seemed to be an essential part of reconciliation with life. Ultimately, reconciling with life meant *alleviating suffering*, making suffering more bearable.

### 3.2 Embodied suffering and loss of meaning in life

The women experienced themselves as exhausted and they frequently suffered from long-lasting pain in different parts of the body, often in joints, the back, or muscles. An overall perception of heavy tiredness or weakness in the body was commonly reported. Experiences of anxiety or periods of meaninglessness were also described:

*In the beginning, I often felt as if I did not want to live, I did not have the strength. There was no will to live. There was no desire and there was no … So, for a long time, I have vacillated between just wanting to give up. I have not gone as far as figuring out how I would do it, I’ve just felt that I do not want to live and had this existential fatigue (6).*

These experiences implied that self-care (e.g., showering), childcare and household chores became overwhelming or insurmountable tasks:

*I could not get out of bed. The days just bled together. My head was just a mess. I could not think clearly. I could not take care of myself. I could not go outside. I walked around in my pajamas all day long. Yes, it was a terrible time. (12).*

The women’s physical suffering varied and could be severe in nature. They sometimes developed neurological symptoms, such as temporary paralysis, fainting, loss of speech or convulsions, all of which could not be explained medically, though they needed specialist care at the hospital. This was expressed as being *weak,* “*could not do anything*, *could hardly walk,” (4)* and *“had to ride in an ambulance.”* As one woman recalled: *“I wasn’t conscious. It was as if I was in a coma, because I could hear everything, but I could not make myself understood and they could not wake me up” (8).* Another woman explained how she was impaired for several years when she *“had convulsions, the head went sideways, the arm might go up” (10)*.

The women reported that their suffering had developed, or at least accelerated, during child rearing years. Mothers of younger children described that their ill-health started during pregnancy, in connection with childbirth or during the first years of being a mother: *“…then, after a few years, that’s when I felt it the most” (11).* Sometimes, signs of ill-health had emerged as early as childhood or adolescence. The women struggled with their tiredness and pain, though some had found ways to alleviate and live with their physical and mental suffering. Two women differed from the others in that their pain could be medically explained.

### 3.3 Relational vulnerability is uncovered in motherhood

#### 3.3.1 The meaning of motherhood

Being a mother was both experienced as an *enrichment in life (1)* but also as a source of strain. Motherhood was described as double-edged: “*…usually, it feels nice to be important, but sometimes it can also be a bit smothering in a way” (5).* Being a mother meant “*love,”* “*joy,”* “*happiness,” “togetherness,”* “*pride”* and, first and foremost, “*meaning*.” It could involve getting in touch with an inner strength and building up self-esteem. One woman described “*how the power of being able to give birth to two children”* made her “*feel so strong” (2)*. For one woman, becoming a mother implied a positive confirmation, helping her to develop a sense of self-esteem that she had not experienced before:

*I am often told I am such a good mother, and you cannot always seek reassurance in that, but it gives me a positive feeling. I feel like I’m getting it right. There is nothing destructive in my life anymore […]. It’s great for my self-esteem. (3).*

To be a mother was also experienced as an intense, life-long commitment that required them to be present and attentive whenever needed. Their own needs were put aside, or disregarded entirely, and time for rest and recovery was scarce. This side of motherhood could be described as “*arduous,” “tough,” “tiring,”* even burdensome and unbearable:

*There have been so many years of their lives where I did not want them (the children) or did not have the strength to care for them and be a mother, because I myself was in such a bad place. You have to do that yourself. […] So I had to take them anyway. (6).*

Sleep became disrupted for many years. The women were concerned when their children went through challenging periods in life, when they were apart from their children or when unforeseen situations arose in their child’s everyday life. Often, the women felt primarily responsible for managing these kinds of challenges and concerns. Two women described their deep concern over whether their prematurely born children would survive and what it meant to care for them, for their siblings at home and how their own needs were put aside:

*When the little one was born and almost died and everything was really hard, I felt that he (the son) had just started kindergarten and everything was so messy in his life anyway, you do not need your mother crying. It’s too much. (1).*

#### 3.3.2 Relational vulnerability in motherhood

Being a mother revealed a need for help and support from others, which was not readily available, leaving them lonely and vulnerable to the daily challenges of a life with children. Thus, the women felt lonely and overburdened by challenges, responsibilities, concerns, and duties connected to motherhood, because they simply *“cannot count on family members for help, there is no help to be found there” (3).* In the children’s upbringing, the women often felt that the main responsibility for the children rested solely on their shoulders*: “*…*I basically single-handedly managed the family” (10).* Either the partner worked long hours and traveled frequently for work or he was simply not engaged in parenthood. These responsibilities were often related to practicalities in the everyday life with raising children, their education, emotional responsiveness toward the children and conveying values for life:

*I was very lonely in the relationship I had with their father for eleven years. I mean, he mostly worked so I was the only one who … I took the kids to school and daycare and picked them up and, well, we cooked dinner and then he came home around the time they were going to bed. So, I was kind of just a mom all the time and did not have many friends. I just had my kids. (13).*

Separating from a partner implied tough years of bringing up their children in shared childcare arrangements or completely on their own, often under economically strained circumstances. Practical and emotional support from their own families or mother, in particular, were scarce. Being emotionally and/or practically supported by their own parents, the extended family or friends was experienced as a source of relief: *“It was a great relief to tie such strong bonds, to always have someone who knew my children so well” (10).*

### 3.4 Relational vulnerability as a breeding ground for strong ideals of motherhood

#### 3.4.1 Relational vulnerability from early on

Having experienced a sheltered childhood was a rare experience: it was more likely that childhood was a difficult experience. The women shared how they had grown up with emotionally absent parents, with a single mother, under complex circumstances, or with parents who suffered from alcoholism. Thus, early relations in life had been unreliable or even insecure, leaving the women vulnerable on a relational level. Overall, the women navigated through life with scarce support from others. This turned out to be a hindrance to health later in life, in particular during motherhood when they were more likely to become dependent on others for care and support. Having suffered from scarce emotional support as a child or having had emotionally instable parents could still be painful:

*I think I was always materially secure, if I think back to when I was young. […] But I did not have the mental and emotional security. And I think that type of security is really important. I get emotional when I bring it up.” (6).*

Therefore, entering adulthood had often been accompanied by strained or insecure relationships with their own parents. Disregarding one’s own needs and managing life without the help and support of others was a reality from an early age: *“… I had to manage on my own a lot, from the start, you might say (14).* The women were vulnerable in relation to their earliest and closest relationships in life and, consequently, also in relation to themselves as human beings and mothers with own needs. Becoming a mother of a son then implied, for this woman that she *“stopped being a human being in some way. I became an instrument for his survival”* (5). During adolescence and adulthood, the women recalled that they searched for confirmation in promiscuous sexual behavior or had tried to temporarily fill a sense of relational emptiness with drugs or alcohol. As adults, the women tended to enter into romantic relationships in which they often felt lonely. The partner could even be a severe strain or threat to the health of the woman. The women who had been exposed to mental and physical abuse in their relationships had separated from their partners after many years. Friendships were often perceived as tiresome. Occasionally, sheltered relationships or friendships of mutual trust that were perceived as meaningful to the women’s own health were mentioned.

#### 3.4.2 Emerging ideals of motherhood

Idealistic views, which were deeply rooted in early life experiences, could become a breeding ground for suffering in motherhood. Being a mother meant offering their children an upbringing that was much different and more sheltered than their own. The women strived to achieve a mother ideal that had emerged from a few happy – but often difficult – childhood experiences. One woman felt *“great anger and sadness over how my life was, and I naturally want to do the exact opposite for my children, in every way” (2).*

Women who reported feeling unsupported by their own parents during childhood described how they actively (had) supported their children in their education, worked to instill good values, tried out various activities, and tried to give them a strong foundation for life, “*something to fall back on” (1).* The women emphasized that it was “*so important that they (the children) have the best life possible” (2),* to offer their children what they themselves painfully had missed out on as a child. The women who reported feeling emotionally neglected when growing up became very committed and attentive to their children:

*It is important to me to be present. When I was a child, I remember it was so obvious when the adults did not listen to you. When they were not interested in what you had to say. You had to yell to get attention, and I do not want my children to have to do that. (3).*

In childhood, the women often “*had no personal space. I just wanted to give my children what I myself did not get: presence” (12).* One woman remembered how as a child, she had been” *very angry but not allowed to express it. That was absolutely not allowed” (2).* Thus, it became central to the woman to give her own daughter space to express herself by singing in a choir, dancing or painting. In tears, she explained:” *I just want her to be able to express everything she has inside” (2).*

#### 3.4.3 Living up to ideals of motherhood gives rise to inner conflicts

The women strived hard to accomplish these more or less conscious ideals, which could come at the cost of their own health and result in suffering. Living up to the ideals of a *normal*, *good* or even *perfect mother,* who offers her children a sheltered upbringing, resulted in inner conflict between the mothers’ own needs and the mother ideal. When they were unable to constantly live up to the ideal of the patient and attentive mother, the women felt insufficient or that they had failed as mothers, which could feel “*terrible” (2).* When their own behavior did not match that of the ideal mother, this could reinforce inner conflicts:

*I might have some ideal that I am somehow trying to find and live up to – but there is a lot of anxiety tied to that, too, when you see that you cannot quite manage to live up to that ideal. Constantly worried about not being enough as a mother. (2).*

Due to her rheumatic pain, one woman was unable to engage with her children in many activities outside the home, “*things everyone seem to be doing” (3)* and suffered from feelings of guilt that she was not the mother she wanted to be. She felt that she had to compensate for this by *“being present and doing as much as I can anyway. But I feel that is the hardest thing, not having as much energy as I would like” (3).* In her everyday life with her children, she felt forced to set her own needs aside. Being with her children implied being” *much more in pain […] because you never have time to recover when you have a two- and five-year-old at home. There is constant running around and fixing and helping, back and forth. My body is exhausted when I am caring for them” (3).*

Striving to create a classic nuclear family despite abuse from her partner led to suffering over many years: “*Being alone with the children”* most of the time, one woman remembered: *“… I just sucked it up. Mm, whereof this dedication, that I am going to handle this, my children are not going to suffer because of this” (12).* Trying to live a normal family life on the outside had been harmful to her health, “*keeping up this façade, that has probably taken quite a toll” (12).* The women rarely felt that they could be or had been the mother they wanted to be without suffering from overwhelming inner conflicts.

### 3.5 Carrying adverse life experiences and inner conflicts in loneliness become an unbearable burden

Adverse life experiences were common among the women, and they often carried these memories alone. Existential feelings of loneliness, anxiety, guilt, sorrow, or anger could be overwhelming. Carrying these feelings and difficult experiences for years or even decades was both painful and exhausting and became an unbearable burden. Some women had been able to integrate difficult encounters in their lives, whereas others suffered under the burden.

The women described how interactions with their own children could awaken traumatic memories from their childhood, how experiences “*are triggered when you have children yourself” (1).* In their everyday lives with their children, the women reported that they could become overwhelmed by feelings of anxiety, anger or extreme tiredness:

*It was during my son’s childhood when my own Pandora’s box opened and I became very ill. It was as if I just wanted to lie under the covers and not get out, I just wanted to lie there. But then I had to get up because I had a child. (9).*

Another woman explained:

*I remember when I was four or five years old and helped her (the mother) when she was drunk and all that. Helped her clean off vomit, put her in bed and everything. Now I look at my own children, they are so small and so vulnerable, and I cannot imagine that such things happen when you are that young. It triggers a lot in me. (2).*

The women who had been exposed to abuse in their relationships felt guilty about not having been able to protect their children from difficult experiences. In parallel with being a mother, the women often took care of their fragile parents and/or siblings with mental illness during parts of their life. This was an exhausting burden that provoked feelings of concern, anger, guilt or tiredness: “*I have kept everyone under my wings for so long that I cannot do it anymore” (14).*

The women also reported exceptional periods of struggle in life, which were intertwined with motherhood. One woman had been homeless; now she deeply mourned “*all these years lost” (13)* with her children and the lost contact with one of her children. The woman had feelings of having let down her children; she felt “*worthless”* and “*blamed”* herself. Another woman shared how having been addicted to alcohol during her children’s upbringing meant that she has struggled with feelings of irresponsibility and guilt*: “I completely ignored the girls. But they still managed quite well. It was very irresponsible of me because I drank in the daytime, too, and was very irresponsible” (6).* Now, she mourned losing out on an initial loving relationship with her first-born daughter, who had now become more distant. The women were often left alone with their heavy life burden which further contributed to feelings of suffering.

### 3.6 An existential shelter makes suffering bearable and helps women reconcile with life

Living in a secure home or being embedded in supportive relationships helped the women let go of previous experiences and made their suffering more bearable. Leaving adverse life experiences and ideals behind, “*dealt with this a long time ago,” (14)* led to reconciliation with life and an altered relationship with themselves. Those women who had reconciled with life perceived their home as a place where they could express themselves and their suffering; they felt existentially sheltered. Nevertheless, letting go of abilities or experiences had been a painful process: *“It’s been painful. It cost a lot” (12).* It involved going through a period of mourning that eventually opened the door to reconciliation with life. The women expressed that they had to find reconciliation entirely on their own, something they could “*not place on someone else’s shoulders” (9)*, because it is a matter of the self:

*At first, there’s grief, as if you are losing a friend or a human or an animal or something, that it’s a sorrow you cannot bear, you have lost your body to something, and I think you need to process that grief, like regular grief. You need to work through it and think, that, yes, there are other values, it’s not the end of the world and all that. You need to come to that conclusion yourself. (7).*

Being embedded in authentic relationships may have facilitated the ability of these women to let go of experiences and ideals and to go through this process of reconciliation. The women who had previously lacked a relational embeddedness had started to build up an existential shelter in which they could be themselves. They had left relationships that were harmful to their health: The women had temporarily broken off contacts with their own mothers, had broken away from harmful relationships, put friendships that drained their energy on hold, or had let go of responsibilities and ideals:

*…maybe you are meant to break free. To realize that … I mean, I am held anyway. I do not need my family to be held. But it’s a bit difficult to find that, that sense of security, that life carried me. (6).*

Being more confident in their relationships with themselves, starting to take their own needs seriously, and expressing themselves helped the women build up an existential shelter. They felt less exhausted and alleviated pain; their suffering had become more bearable. Women who had reconciled with life, or who were on their way to reconciliation, felt enriched, *“had found inner peace” (12)* or were aware of a changed relationship with themselves: “*I mean, I’ve become a completely different person since I became ill. Going from being this exuberant, being in the spotlight and entertaining an entire party and having a hundred irons in the fire and … To being a calm person” (8).* In finding reconciliation, the women lived through a process of an altering and growing self, feeling that this was a necessary and lifelong development to eventually re-balance their health:” *… to become the person you want to be for yourself and for your family and for the people you love around you. It’s up and down. And it’s something you will need to continue reflecting on for the rest of your life” (5).*

Regardless of where in the reconciliation process the women were, they all felt that being a mother had carried them through times of suffering; it had been a *“driving force” (9)*. One woman who had struggled intensely with being a mother reflected:” *… if they had not been born into this world through me, I would not be the person I am today, because I am somewhere in a more beautiful place inside than I ever was. I would not be if it were not for them” (6).* Being a mother meant a lifelong commitment and being vulnerable, but it also meant that their own children became part of an existential shelter, particularly when they were grown adults: *“I curled into his (the son’s) calm when I was sad when my mother died. […] I lay on the floor in his room, in his calm” (1).* Another woman gratefully described:” *I can come home to my daughter and, ‘Mom, you are staying for dinner, right? Take a seat on the couch, rest a while, I’ll take care of everything’” (12).*

## 4 Discussion

In this study, a phenomenological approach was applied to seek a deeper understanding of how motherhood, as one aspect of gender identity, interacts with pain and exhaustion. We identified relational vulnerability as a key phenomenon in the experience of pain and exhaustion among women and as a threat to women’s health during motherhood.

Unreliable or even harmful relationships that started in childhood were common, and this pattern of relational vulnerability had most often followed the women into motherhood: The women mostly carried out parental responsibilities without the support they needed, and the demands of motherhood were often perceived as insurmountable. The observed phenomenon of relational vulnerability seems to be closely connected to research on adverse childhood experiences and attachment. Adverse childhood experiences are known to be associated with long-lasting pain in adult life but have also been shown to be connected to mood disorders such as major depression (Antoniou et al., 2023; Bussières et al., 2023). On the other hand, secure attachment—both early attachment or attachment earned later in life—is considered to strengthen an individual’s resilience in terms of positive adaptation and encountering adversity (Darling Rasmussen et al., 2019). Consequently, reliable relationships have been suggested to be central in facilitating resilience (Darling Rasmussen et al., 2019). Similarly, reliable relationships that women can turn to for support to deal with parental challenges and strains have been found to be significant for women’s well-being and ability to combine working life with motherhood (Luthar and Ciciolla, 2015; Dow, 2016; Luthar et al., 2019). In a recent longitudinal cohort study (Samulowitz et al., 2023), it was found that women with weak social support were at a higher risk to develop pain conditions, both compared with women with strong social support and compared with men. In particular, low practical support was found to be an important predictor of pain for women (Samulowitz et al., 2023). Similarly, to these findings, mothers living with exhaustion and pain were found to perceive particularly less social support from family, friends, and significant others compared to their healthy counterparts. Furthermore, the less supported these mothers felt by significant others, the more suffering they reported (Gebhardt et al., 2021). Seen from an evolutionary perspective, the significance of social embeddedness for mothers is nothing new: raising offspring has never been a lonely task for our human ancestors. Rather the opposite was the case: through the development of empathy, a supportive environment and cooperative breeding was developed, which facilitated survival (Hrdy, 2011). In other words, the evolution of our own species has been dependent on mothers’ ability to connect with others and the received support of others in caring for offspring (Hrdy, 2011). Thus, it does not come as a surprise that over the long term, the relational vulnerability and resulting feelings of loneliness in family life became exhausting to the mothers in our study. However, the significance of supportive relations during motherhood is rarely acknowledged regarding pain and exhaustion.

Although more than half of the mothers in our study were living together with a partner, almost all women expressed loneliness in caring responsibilities for children and/or close relatives. According to a population study of cohabiting and employed men and women in Sweden, more women (41.3–61.0%) than men (4.2–5.1%) report to be mainly responsible for domestic work, but an association with sickness absence was not found (Staland-Nyman et al., 2021). Similarly, a register-based study of Swedish parents (Angelov et al., 2020) concluded that ill-health (measured as in-patient hospital care) could not explain the gender gap in sickness absence among parents. Instead, it is suggested that women balance commitments to family and at work with sickness absence (Angelov et al., 2020). Although these studies have a robust methodology, they may fail to capture ill-health that could be explained by gender-specific challenges complicated by a unique personal history. Many mothers in our study—despite cohabiting—were lonely in a double sense: they shouldered family life alone, and they could be alone with memories of adverse life experiences. These elevated levels of loneliness seemed to damage the women’s health, making them suffer under what we have termed an *unbearable burden* because previous adverse life experiences combined with current life circumstances constituted an overwhelming burden. A term closely connected to this observed phenomenon—“chronic struggle”—has been brought forth by Webster et al. (2023) to underline the various social challenges in life that individuals suffering from pain constantly encounter. Our findings suggest that participating mothers suffered from a stressful life situation and a social pain that may have manifested itself in physical pain. A connection between stress, social pain and physical pain has previously been presented in the scientific literature (Sturgeon and Zautra, 2016; Nijs et al., 2017; Lunde and Sieberg, 2020). Physical and social pain were found to be connected on a neurological level, which possibly could be explained from an evolutionary point of view: in order to survive, an individual without reliable social relationships must be stimulated “to repair the social schism or to seek new sources of support” (Sturgeon and Zautra, 2016, p. 65). From an existential point of view, health “inevitably involves a consideration of the social context” and the interdependence with others because our understanding of life and the self is formed and exists in the relationship with others (Herring, 2016, p. 24). In a similar manner, the philosopher Svenaeus concludes that “[w]e suffer due to our vulnerable bodies and the vulnerable relations we form with other vulnerable persons through our being-in-the-world” (Svenaeus, 2018, p. 144). Taken together, pain and exhaustion could be understood as signs of suffering from being left alone and overburdened with the task of child rearing that never was meant to be a task carried out by one single person.

Nevertheless, the participating women found ways to live life that made their suffering bearable, and some of them were able to reconcile with life. This process was described as painful by the women because they had to let go of relationships, abilities, or ideals that were interwoven with their self and motherhood. In a meta-ethnography on recovery from chronic pain (Toye et al., 2021), a similar process has been described as a healing journey that stretches from being torn apart from the world and oneself to become reconnected with the world and oneself. Notably, the women in our study described this period—despite being painful—as an iterative movement toward something positive, a process we have identified as reconciliation; a new understanding of life and themselves had started to unfold. This observed phenomenon of reconciliation emphasizes the significance of defining health not as a state, but as an ever evolving process in the presence of suffering and vulnerability (Eriksson, 2006; Herring, 2016; Leonardi, 2018). The strength to face one’s vulnerability as human being has been described as a prerequisite to personal growth; a strength that implies being able to respond in a meaningful way to what is given in life (Binder, 2022a). The women who had that strength were able to reconcile with their own life story and to relate to themselves and others in a new way. In the remaining discussion, we address how an *existential shelter* gives the women the strength to reconcile with life.

We defined existential shelter as the availability of authentic relational ties and places in space or time where the women were able to be themselves and where they were cared for themselves. Women with stress-related ill-health have previously been found to experience a need for unconditional beingness to feel well-being (Hörberg et al., 2020), for a place where one can feel sheltered and at home. Samzelius (2020) has highlighted the very necessity of an adequate and safe homeplace where mothers are able to nurture their children. Homelessness has been described as very damaging to the health of mothers (Samzelius, 2020). The experience of being homeless—in a literal but also in a figurative sense—applied to several participating women. The experiences of not-being-at-home and of being alienated have from a phenomenological point of view been pointed out as key elements of suffering (Svenaeus, 2015, 2018). The question is whether the women in our study already from early on in their lives found themselves in a world that was an unhomelike place to live in, which made them experience themselves as alienated from the world and others. Traumatic experiences in life have been considered to awaken feelings of homelessness in life (Binder, 2022a). Such an interpretation would suggest that the women’s pain and exhaustion are expressions of a suffering that may have their origin in a figurative (and in some cases literal) homelessness—a missing shelter in life: The participating women were scarcely nurtured in relationships, a phenomenon that has been highlighted previously among women with pain and/or exhaustion (Arman and Hok, 2016; Arman et al., 2020; Gebhardt et al., 2021). However, women do not just need to be supported in parental responsibilities as outlined above, as caregivers, women need to be cared for, as nurturers to be nurtured, and as mothers to be mothered (Luthar, 2015; Herring, 2016).

Suffering is fundamentally human; to share suffering with others offers a way to relate to oneself and one’s own suffering in a new sense; recognition and compassion play a vital role in such encounters (Binder, 2022b). Therefore, we would like to emphasize the need of a care that helps women build, but also temporarily offers women an existential shelter—a homelike place—from which they then are able to move on in life. The need for a healthcare where women with pain can explore existential questions has previously been emphasized (Andermo et al., 2017). Having one’s experience validated was found to be an important aspect of care that enabled a new understanding of oneself when suffering from chronic pain (Toye et al., 2021). Still, women who are afflicted with this kind of suffering often face the opposite; they feel that they are not taken seriously and sometimes even that they are abandoned by a biomedically oriented healthcare system (Toye et al., 2013, 2021; Arman et al., 2020; Webster et al., 2023). What might be needed, to come to terms with these challenges within healthcare, has been pointed out by Binder (2022a,b): “In a highly medicalized society, we need to reinvent our language for these existential struggles, and the suffering that always accompanies them” (Binder, 2022b, p. 6). Within this study, we strived to contribute to a language that captures central phenomena of existential health within the field of pain and exhaustion.

The feelings of suffering experienced by the women in this study may have been reinforced, perhaps even have developed, during motherhood. But at the same time, motherhood added a tremendous sense of meaning to their lives, making their suffering bearable. This further underlines the complex interaction between women’s health and motherhood. Women who experience exhaustion and pain are at the verge of their limits; nevertheless, they continue to care for their children. As long as motherhood is not recognized in general healthcare encounters as an existential aspect of women’s health and suffering, mothers remain silenced and suffering.

## 5 Limitations

Ricoeur’s interpretation theory (Ricoeur, 1976) has allowed us to discover unfolding, meaningful structures within women’s experiences of pain and exhaustion. These phenomenological structures have helped us build an understanding of women’s health and suffering and the close relationship to motherhood. According to Ricoeur (1981), understanding requires explanation, which we documented in naïve understandings, quotations, and a critical interpretation. However, there is always more than one single interpretation. Considering this fact, we strived, as far as possible, to arrive at the most probable interpretation in discussions with each other. Nevertheless, the presented interpretation should be read with caution and causal conclusions cannot be drawn. Our study is strengthened by the fact that we interviewed each woman twice, giving us the opportunity to confront and discuss emerging interpretations during the interviews. Nevertheless, we would like to acknowledge that our interpretation touches on psychosomatic aspects and that we in no way wish to diminish possible underlying biomedical causes of suffering. The women’s medical diagnoses were not systematically collected because the fact that the women were entitled to a rehabilitation program for long-lasting pain and/or exhaustion (which is paid by Sweden’s social insurance system) was considered as a strong indicator of severe ill-health. However, this lack of information about specific medical diagnoses makes the interpretation of the women’s experiences more difficult to apply to specific patient groups.

In the phenomenological research tradition, rich personal descriptions of experiences rather than data saturation are the focus of data collection (Saunders et al., 2018). The reason is that descriptions of experiences are considered to always remain incomplete. Thus, there will always be nuances of a phenomenon that will not be captured. The phenomenological structures presented in the results unfolded clearly when reading the 27 interview transcripts and corresponding naïve understandings which most closely corresponds to theoretical saturation as described by Saunders et al. (2018). We are aware that there must be nuances in the presented phenomena that remain unobserved, at the same time we are confident that we have sufficiently rich descriptions of experiences to underpin and illustrate identified phenomenological structures. Furthermore, all participants’ experiences are adequately represented both in the interpretative texts as well as directly with quotes.

When studying vulnerable groups, deficit discourses often emerge, in which individuals are characterized by researchers in a negative sense, apparently in terms of illness instead of also focusing on strengths (Mollard et al., 2020). From an ethical point of view, this can be problematic, as a deficit discourse risks reinforcing defective beliefs, both among individuals and healthcare professionals (Mollard et al., 2020). Despite our focus on working out the phenomenon of relational vulnerability, we also focused on women’s strength and ability to reconcile with life, their personal growth, and their ability to consider motherhood as a source of meaning in life.

## 6 Conclusion

Motherhood is an existential part of women’s health and suffering. However, it is scarcely recognized as such in general healthcare encounters. In this study, we have elaborated on how essential an existential understanding of health is when caring for women with long-lasting ill-health, such as pain and exhaustion. An existential understanding of health acknowledges suffering and vulnerability as natural parts of life that can give rise to personal growth. Being a mother means that vulnerabilities are both uncovered and reinforced in a way that can cause suffering that can be close to unbearable. Being vulnerable means being dependent on others. Offering women an existential shelter through caring encounters in which women are invited to reflect on their experienced burdens and demands of motherhood has the potential to help women reconcile with life. In this way, pain and exhaustion among women who are also mothers can be alleviated.

## Data availability statement

The datasets presented in this article are not readily available because data cannot be made publicly available for legal and ethical reasons. Requests to access the datasets should be directed to AG, anja.gebhardt@ki.se.

## Ethics statement

The studies involving humans were approved by Swedish Ethical Review Authority. The studies were conducted in accordance with the local legislation and institutional requirements. The participants provided their written informed consent to participate in this study.

## Author contributions

AG: Conceptualization, Formal analysis, Funding acquisition, Investigation, Methodology, Writing – original draft, Writing – review & editing. SA: Funding acquisition, Supervision, Validation, Writing – review & editing. MA: Funding acquisition, Project administration, Supervision, Validation, Writing – review & editing.
